# Overweight, Thinness, Body Self-Image and Eating Strategies of 2,121 Italian Teenagers.

**DOI:** 10.1100/tsw.2005.96

**Published:** 2005-09-28

**Authors:** Roberta Guarino, Alberto Pellai, Luca Bassoli, Mario Cozzi, Maria Angela Di Sanzo, Daniela Campra, Andrea Guala

**Affiliations:** ^1^ SOC di Pediatria, Ospedale SS Pietro e Paolo, Borgosesia, ASL 11-Regione Piemonte, Italy; ^2^ Istituto di Igiene e Medicina Preventiva, IRCCS Ospedale Maggiore, Milano, Italy; ^3^ Centro di Riabilitazione–Psicomotricità, ASL 1 Tortora–Regione Basilicata, Italy

**Keywords:** adolescence, child health, development, body self-image, eating disorders, Italy

## Abstract

This study describes the prevalence rate of overweight and thinness in a population of teens living in two different areas of Italy and explores the body self-image perception and unhealthy eating behaviours and strategies to lose weight. A questionnaire was administered to a sample of 2,121 teenage students (1,084 males; 1,037 females). Results showed that teen females and males build and perceive their body images in very different ways. Most of the overall sample perceived their weight as normal, while a relevant 31.6% defined themselves as overweight and another 4.4% as heavily overweight. Analysis based on BMI (calculated through self-referred weight and height) showed that only 9.2% of our sample could be considered overweight and 1,7% obese. Most of female teen students (485 out of 1,037) were trying to lose weight, demonstrating that strategies to lose weight were undertaken also by girls perceiving themsleves as normal in relation to body weight; 46.8% girls were using strategies to lose weight compared with 21.9% boys. These strategies included very problematic behaviours like self-induced vomiting (3.3% F vs. 1.7% M) and dieting pills (2.8% F vs. 1.5% M) undertaken along with more usual thinning strategies like dieting and exercising. Girls were more prone than boys to exercise as a way to lose weight (41% vs. 31.7%). This study showed that there is a deep gap between actual weight and perceived body-image and weight. This study is one of the first of this kind in Italy and calls for primary prevention and health education programs aimed at improving teen body-image as a strategy to reduce the eating disorder epidemics spreading among young people.

## INTRODUCTION

The number of teenagers who perceive themselves as overweight and adopt unhealthy strategies to lose weight is constantly increasing, as is clearly evident by some recent epidemiological surveys carried out in Italy[1,2]. Too often, teenage slimming is obtained through problematic eating habits, overexercising, purging, and vomiting. These risk behaviors often bring teenagers to reach an unsuitable body weight or one that is even dangerous for their health[1,3]. Data from the U.S. Centers for Disease Control and Prevention (CDC) indicated that the number of American teenagers trying to lose weight has been constantly increasing over the past 10 years and the percentage of young girls under dietary restraint has grown from 43.5 to 59.5%, while the percentage of boys is from 15.5 up to 26%[2,3].

The problem seems to be related specifically to the current cultural attitude, which attaches thinness as a valuable esthetic canon capable of ensuring a successful life[4]. The ideal body self-image of the adolescent derives, in fact, from identification with someone else's body due to mass-media influence and from the cultural environment that glorifies beauty and physical shape. For example, photos shown in fashion magazines have a deep impact on girls' body size and weight perception, as can be seen from interviews of 548 female teenagers (10–17 years), which showed that 69% reported that they were influenced in their ideal body self-image and 47% reported that they wished to lose weight[5].

A number of young girls subject their bodies to significant weight-loss efforts at a particular time of growth when their body specifically requires enriching and strengthening, with a negative impact on their pubertal functional levels (organic, psychological, and social). Excessive concern with weight and indiscriminate dietary restraint might have a potential negative impact on mental and physical health and could easily compromise learning ability. A poor nutritional status may have negative effects on cognitive faculties and a bad influence on scholastic performances and social acceptance. A reduced calorie intake of some nutritional principles leads, in fact, to memory diminution, reduced concentration power, and an increase in irritability. A poorly balanced diet in students finally translates into inactivity, indifference, and reduced capacity to interrelate with the environment[6]. A deliberate and prolonged dietary restraint might result in a lack of iron, provoking weariness, shorter attention span, lessened capacities in physical and mental activities, weaker resistance to infections as well as a delay in growth and sexual maturity. American surveys reported that 2–4% of teenaged girls have evidence of iron deficiency[7].

Weight concern might also influence the adoption of other at-risk behaviors. It has been noticed that the onset of cigarette smoking is higher among those adolescents on a diet or who are excessively worried about their weight compared with adolescents satisfied with their body shape and weight. Female high school students, white girls and smoke addicts, reported that they use cigarette smoking to keep their weight and appetite under control[8].

The present study was carried out to describe the prevalence rate of unhealthy and risk behaviors in teenage Italian students living in two country areas. The research aimed also at revealing how teens value their actual body image and weight in relation to their BMI (calculated through self-referred weight and height).

## METHODS

The survey was carried out in March–April 2003 in two mountain areas with similar sociodemographic characteristics located in Valsesia (Regione Piemonte, Northern Italy) and in Lagonegro (Regione Basilicata, Southern Italy). The survey's tool was an anonymous self-reported questionnaire filled out by all adolescents attending high schools (from I- to V-year courses). The research was carried out in the same month in both areas. The ages ranged between 14–29 years, but the final sample was obtained after eliminating students 20 years or older (since they represented only 1.5% of the total sample).

Participants filled in their questionnaire at school, during a morning class. Time available to complete the survey was half an hour. The survey tool was the Italian version of the YRBSS (Youth Risk Behavior Survey System) used since 1989 by the CDC to monitor at-risk behaviors among teenagers[9]. The YRBSS is currently employed in U.S. surveys with high school students to monitor preventive interventions[10,11,12]. More specifically, the YRBSS is used to analyze eight at-risk behaviors that play an important role in determining the health profile and social aspects of the teen population. Our research used only some sections of the global YRBSS questionnaire, in particular those dealing with body weight, body self-image, and strategy to lose or control weight with a total of 12 multiple-choice questions. All students were asked to report their sex, age, weight, and height. BMI (Body Mass Index) was calculated by putting in relation weight (expressed in kilograms) with squared height in meters (BMI = $kg/m^2$). The phenotype classification by BMI size ranges was corrected by age and by Italian data[13,14]: underweight (males: <16 at 14 years, <18 at 19 years; females: <16.5 at 14 years, <17.5 at 19 years), average weight (males: 16–24 at 14 years, 18–24.9 at 19 years; females: 16.5–24.9 at 14 years, 17.5–24.9 at 19 years), overweight (males: 24.1–29.5 at 14 years, 25–29.9 at 19 years; females: 25–29.9), obesity (males: 29.6 and more at 14 years, 30 and more at 19 years; females: 30 and more).

Approval for the study was obtained from the ethics committee of the Regione Piemonte and Regione Basilicata. The data collected were entered into and elaborated by EpiInfo6, a word-processing database and statistic program edited by the CDC.

## RESULTS

Seven secondary schools participated in the study in Valsesia with a total number of 1,950 collected questionnaires and four schools in Lagonegro with a total number of 937 collected questionnaires. Of the 2,887 questionnaires, 2,158 (75% response rate, 1,350 from Valsesia and 808 from Lagonegro) were available for data analysis. Of the returned questionnaires, 25% were considered invalid due to blank or unanswered questions. Distribution by age and gender is provided in Table 1. On all the items of the two geographical samples, statistical analysis was performed using the Chi Square Test, but no statistical significance ($p \leq 0{,}05$) was found to differentiate some characteristics of the two subsamples. For this reason, this paper presents results obtained through analysis of a unique database, containing both Valsesia's and Lagonegro's data.

**TABLE 1**
**Participants Age and Gender**

| Age (Years) | 14 | 15 | 16 | 17 | 18 | 19 | Total |
|---|---|---|---|---|---|---|---|
| **Female** | 113<br>10.9% | 220<br>21.3% | 196<br>18.8% | 204<br>19.7% | 235<br>22.7% | 69<br>6.6% | 1037<br>48.9% |
| **Male** | 159<br>14.7% | 292<br>26.9% | 242<br>22.3% | 186<br>17.1% | 138<br>12.8% | 67<br>6.2% | 1084<br>51.1% |
| **Total** | 272<br>12.8% | 512<br>24.1% | 438<br>20.7% | 390<br>18.4% | 373<br>17.6% | 136<br>6.4% | 2121 |

Table 2 indicates the participants' actual weight, on the basis of BMI stratifications (underweight, average weight, overweight, and obese), calculated on the basis of their self-reported measure, for age class.

Body weight perception was assessed through the question "How would you define your weight?" The given choices were the following: extremely underweight, a little underweight, average, a little overweight, obese**.** Table 3 reports self-perceived body weight, stratified by BMI. In our sample, on the basis of BMI values, 194 students can be considered actually "overweight" (9.1%) and 37 "obese" (1.7%). Perception of being overweight and obese interests a much larger group: in fact, 670 (31.6%) consider themselves as "overweight" and 93 (4.4%) as "obese". The perception of being overweight or obese (36%) resulted in actions or attitudes aimed at reducing body weight (34.1%) (Table 4). Most female respondents reported that they were currently trying to lose weight, while the percentage of males who reported that they followed some weight-losing strategy was consistently lower, the ratio between females and males being nearly 2:1. Female responders trying to gain weight were only one-third of the

**TABLE 2**
**Participant Actual Body Size by BMI Classes, Corrected for Age and Italian Data[8,9]**

| Age (Years) | Number | Underweight | Average | Overweight | Obese |
|---|---|---|---|---|---|
| 14 | 272 | 170 | 75 | 24 | 3 |
| 15 | 512 | 300 | 174 | 35 | 3 |
| 16 | 438 | 238 | 146 | 41 | 13 |
| 17 | 390 | 178 | 161 | 42 | 9 |
| 18 | 373 | 199 | 134 | 35 | 5 |
| 19 | 136 | 58 | 57 | 17 | 4 |
| Total | 2,121 | 1,143<br>53.9% | 747<br>35.3% | 194<br>9.1% | 37<br>1.7% |

**TABLE 3**
**Perception of Own Body Self-Image in Relation with BMI, Adjusted for Age and Italian Standard[8,9]**

| Perception | Number (%) | M | F | BMI | M | F |
|---|---|---|---|---|---|---|
| Underweight | | | | | | |
| Extremely | 34 (1.6%) | 25 | 9 | 1,143 (53.9%) | 488 | 655 |
| A little | 275 (13%) | 192 | 83 | | | |
| Normal weight | 1,040 (49%) | 573 | 467 | 747 (35.2%) | 422 | 325 |
| Overweight | 670 (31.6%) | 252 | 418 | 194 (9.2%) | 144 | 50 |
| Obese | 93 (4.4%) | 37 | 56 | 37 (1.7%) | 30 | 7 |
| No answer | 9 (0.4%) | 5 | 4 | — | — | — |
| Total | 2,121 | 1,084 | 1,037 | 2,121 | 1,084 | 1,037 |

**TABLE 4**
**Desire to Lose/Maintain/Increase Weight Among 2,121 Teens**

| | M<br>1,084 | % | F<br>1,037 | % | Total<br>2,121 | % |
|---|---|---|---|---|---|---|
| I'm trying to lose weight | 238 | 21.9% | 485 | 46.8% | 723 | 34.1% |
| Keeping my weight | 695 | 64.2% | 510 | 49.2% | 1,205 | 56.8% |
| Trying to improve weight | 151 | 13.9% | 42 | 4% | 193 | 9.1% |

corresponding percentage of males. The percentages of female and male respondents trying to maintain the same weight were not statistically different. Sports activities were the most employed weight-controlling strategy, followed by slimming diets in general, self-induced vomiting, and use of slimming tablets or remedies. The slimming or weight-controlling strategies and their frequencies by gender are presented in Table 5. Some students combine more than one strategy to lose weight in an unhealthy way: in fact, among those who declared the practice of self-induced vomiting (n = 53, 34 females, 19 males), 15 used also slimming tablets or remedies (9 females, 6 males).

**TABLE 5**
**Slimming Strategies Adopted by Teens Dissatisfied with their Weight**

| | F | % | M | % | Numbers | % |
|---|---|---|---|---|---|---|
| Sporting activities | 425 | 41 | 344 | 31.7 | 769 | 36.2 |
| Slimming diet | 260 | 25 | 78 | 7.2 | 338 | 15.9 |
| Self-induced vomiting | 34 | 3.3 | 19 | 1.7 | 53 | 2.5 |
| Medicine - remedies | 29 | 2.8 | 16 | 1.5 | 45 | 2.1 |

Numbers refer to strategies and not to individuals, considering that some subjects adopt more than one strategy.

Actual BMI was not related with strategies employed to lose weight. Among those who practiced self-induced vomiting (n = 53) only four were obese and six overweight, while eight could even be considered underweight, on the basis of their BMI. Among those using diet pills (n = 45), two were obese, six overweight, and nine underweight.

The strategies employed to lose weight were homogeneously distributed in all age classes. Only in a class group from a school in Valsesia was an anomalous situation put in evidence: in this class, a relevant group of six students (5 females, 1 male), aged 16 years, were employing both self-induced vomiting and diet pills to lose weight.

## DISCUSSION

This survey showed how different Italian teen boys and girls rate and perceive their body self-image, as clearly pointed out by the 2:1 ratio between females and males with overweight/obese body perception. Though most students in our sample perceived themselves with “normal weight”, the percentages of those perceiving themselves as “a little overweight” (31.6%) or “extremely overweight” (4.4%) appeared quite relevant.

The adoption of weight-loss strategies was reported by almost half of the female sample enrolled in this survey (485 out of 1,037; 46,8%), though almost everybody was ranked as normal through their BMI. Of the female participants who perceived themselves to be “overweight” (418 out of the 1,037 interviewed girls; 40,3%), only 51 were actually overweight (4.9%) according to their BMI. As far as male participants were concerned, those who perceived themselves to be “overweight” were 252 (23.2% of the 1,084 interviewed boys) vs. 143 (13.2%) really ranking in the overweight BMI category. These data show that girls were more likely to have a distorted body self-image, which is in accordance with the findings of others[1,15,16,17]. Regarding weight-loss strategies, we found data consistent with those reported for American teenagers[3].

The percentage of teenage girls reporting self-induced vomiting after eating was 3.3%, thus indicating the presence of at least one student for each high school class suffering from consistent eating disturbances, which might easily develop into clear and serious eating disorders[1,7,15,17]. The number of females adopting weight-loss strategies was also higher than males. In addition, the same subjects often reported to follow more than one slimming strategy to lose weight.

Due to these findings, the research staff planned and implemented a prevention plan, aimed at reaching the most vulnerable classes, attended by clusters of at-risk adolescents, and discussing the problem of attitudes predisposing to eating disorders. This program took place in Valsesia and brought professionals (a psychologist and nutritionist) into five of the seven III-year classes participating in the current study, for a total of 187 teenaged students (75% of them were males and 25% females).

The educational intervention consisted of two meetings of 1 h each, planned and held with the purpose to clearly explain to the students the harmful effects of unsuitable weight-loss strategies, as well as to point out actions to prevent eating disorders.

## CONCLUSION

The problem extent, as defined step by step by the current study findings, is certainly wide, although not directly evident, and needs proper attention both at school and within the family. Every educational effort to prevent, recognize, and treat the problem of disturbed eating attitudes must be encouraged.

Specifically, school health educators and school health service personnel might be of great help in providing the students with the possibility of dealing with the matter at the class level, in encouraging adolescents to recognize their uneasiness and openly communicate it, to teach them a critical way of "reading" the mass-media messages, to address them with basic nutrition information, to explain the risks of a restricted caloric intake as well as the benefits of a well-balanced diet meeting energy needs for growth and development.

School is certainly an appropriate environment to recognize the problem for early diagnosis and easy resolution, but it is above all the most suitable place to provide adolescents with sound foundations for their adult being and self-esteem building, so that they might become aware of their potential as well as of the pressure constantly exerted on them by the prevailing culture. In addition, we firmly believe that any specific preventive actions held in the school context must be extended to the population of primary and lower secondary schools. Although the incidence of eating disturbances reaches its highest peak in puberty, certain attitudes towards food — as well as the way of perceiving one's body self-image — are consolidated at an earlier age[15].

## LIMITATIONS

The current survey was based on self-reported weight and height assessments, which can be seen as a limitation and might translate in a lower level of accuracy, but this way we avoided long procedures of height and weight measurements with a consequent saving of money, time, and human resources. In addition, the self-reported method, extensively employed in numerous scientific studies published in the literature, was noninvasive[19,20] since it prevented responders with specific weight problems (seriously overweight or underweight) from being subjected to measurements at school. Another limitation is that adolescents suffering from eating disorders tend to deny their condition, which results in under-reporting of abnormal eating patterns[21].

## ACKNOWLEDGMENT

We wish to thank Dr. G. Abelli at SIAN, Preventive Department, ASL 11 Regione Piemonte and Dr. F. Galetti at SOC of Psychology, ASL 11, Regione Piemonte, for their cooperation.

## BIOSKETCHES

**Roberta Guarino, MD,** SOC di Pediatria, Ospedale SS Pietro e Paolo, Borgosesia, ASL 11 – Regione Piemonte, Italia.

**Alberto Pellai, MD**, Istituto di Igiene e Medicina Preventiva, IRCCS Ospedale Maggiore, Milano, Italia.

**Luca Bassoli, MD**, Istituto di Igiene e Medicina Preventiva, IRCCS Ospedale Maggiore, Milano, Italia.

**Mario Cozzi, PhD**, SOC di Pediatria, Ospedale SS Pietro e Paolo, Borgosesia, ASL 11 – Regione Piemonte, Italia.

**Maria Angela Di Sanzo, PhD**, Centro di Riabilitazione – Psicomotricità, ASL 1 Tortora – Regione Basilicata, Italia.

**Daniela Campra, MD**, SOC di Pediatria, Ospedale SS Pietro e Paolo, Borgosesia, ASL 11 – Regione Piemonte , Italia.

**Andrea Guala, MD,** SOC di Pediatria, Ospedale "SS Pietro e Paolo", Piazzale Lora n. 1, 13011 Borgosesia, ASL 11- Regione Piemonte, Italy. Tel: 0163/203111; Fax: 0163/203267. E-mail: pediatria.borgosesia@asl11.piemonte.it
